# Serum C1q as a Novel Predictive Biomarker for Postoperative Delirium After Acute Type A Aortic Dissection

**DOI:** 10.1002/iid3.70500

**Published:** 2026-09-08

**Authors:** Jianing Wang, Yi Jiang, Wenxue Liu, Yapeng Wang, Shan Lu, Jiawei Zhu, Xiujuan Cai, Yuhuan Tian, Yongqing Cheng, Dongjin Wang, Yunxing Xue

**Affiliations:** ^1^ Department of Thoracic and Cardiovascular Surgery, Nanjing Drum Tower Hospital, Chinese Academy of Medical Sciences & Peking Union Medical College Peking Union Medical College Graduate School Nanjing China; ^2^ Department of Thoracic and Cardiovascular Surgery, Nanjing Drum Tower Hospital The Affiliated Hospital of Nanjing University Medical School Nanjing China; ^3^ Institute of Cardiothoracic Vascular Disease Nanjing University Nanjing China; ^4^ Department of Thoracic and Cardiovascular Surgery Nanjing Drum Tower Hospital Clinical College of Nanjing Medical University Nanjing China; ^5^ Department of Thoracic and Cardiovascular Surgery Nanjing Drum Tower Hospital Clinical College of Nanjing University of Chinese Medicine Nanjing China

**Keywords:** acute aortic dissection, biomarker, C1q, delirium

## Abstract

**Background:**

This study aimed to evaluate the predictive value of C1q (complement activation regulatory factor) for the occurrence of postoperative delirium (POD) following surgery for acute aortic dissection.

**Methods:**

We prospectively included patients undergoing surgery for aortic dissection. Serum levels of C1q were measured preoperatively and on the first postoperative day. POD was assessed daily postoperatively using the confusion assessment method (CAM) or the confusion assessment method for the intensive care unit (CAM‐ICU) for patients in the intensive care unit (ICU). Statistical analyses included the Pearson correlation coefficient and multivariate logistic regression to evaluate the association between C1q levels and POD.

**Results:**

A total of 67 patients were enrolled in the study. Serum C1q levels were significantly higher in the Delirium group compared to the non‐Delirium group both preoperatively and postoperatively. Univariate logistic analysis revealed that preoperative serum C1q levels were predictive of POD (OR = 1.379, 95% CI = 1.176–1.683, *p* < 0.001). The area under the curve (AUC) for preoperative serum C1q was 0.788 (95% CI = 0.671–0.880).

**Conclusions:**

C1q is a potential biomarker for predicting POD in patients undergoing aortic dissection surgery. These findings suggest that C1q may be valuable for early diagnose and intervention strategies for POD.

**Abbreviations**
AAD: acute type A aortic dissection
AUC: area under the curve
CAM‐ICU: confusion assessment method for the intensive care unit
ICU: intensive care unit
POD: postoperative delirium
RASS: Richmond agitation‐sedation score
ROC: receiver operating characteristic
SAE: sepsis associated encephalopathy

## 1 Introduction

Acute type A aortic dissection (aTAAD) is a critical cardiovascular emergency with high mortality and morbidity [1]. This condition often leads to the formation of true and false lumens within the aorta, resulting in diminished blood pressure in the true lumen, malperfusion of arterial branches, and potential compromise of cerebral perfusion [2]. The exigency of aTAAD mandates immediate medical intervention due to its potential for rupture, and the process of hypothermic circulatory arrest during surgery may also contribute to postoperative neurological complications [3].

Postoperative delirium (POD) constitutes a prevalent neurological complication in patients undergoing aortic dissection surgery [4]. This transient neuropsychiatric disorder manifests as acute confusion accompanied by fluctuating levels of consciousness. POD has been implicated in prolonging hospital stays, elevating mortality rates, and precipitating permanent neurological dysfunction (PND) in some cases [5]. The underlying pathophysiology of POD is multifaceted and has been linked to ischemia‐reperfusion injury, inflammatory responses, neurotransmitter imbalances, and endothelial dysfunction [6]. Current therapeutic interventions for POD primarily emphasize comprehensive care, with pharmacotherapy serving as the cornerstone. Dexmedetomidine, in particular, is administered to mitigate agitated delirium among patients [7, 8]. However, the efficacy of antipsychotics in preventing or treating delirium remains unproven [9]. Nonetheless, precise preventive measures remain elusive. Consequently, vigilant surveillance and thorough evaluation of neurological function in patients with aTAAD are of utmost importance.

Existing biomarkers, such as neuron‐specific enolase (NSE) and S100β, exhibit certain limitations in predicting POD, highlighting the urgent need for an aTAAD‐specific predictive tool [10]. The pathophysiology of delirium, including the molecular pathways that confer brain vulnerability, remains incompletely understood. Complement factor C1q, a crucial initiator of the classical complement pathway, is intimately associated with various chronic inflammatory diseases [11]. Structurally, C1q is a complex glycoprotein comprising 18 polypeptide chains [12], with its C‐terminal spherical head region facilitating the recognition of diverse molecular structures and its N‐terminal collagen tail orchestrating immune mechanisms.

As a pivotal component of the classical complement pathway, C1q may regulate collagen‐induced platelet activation, reactive oxygen species (ROS) production, and related white blood cell recruitment during vascular injury [13]. Notably, C1q has been reported to correlate with delirium in sepsis‐associated encephalopathy [14]. Based on these findings, we hypothesize that C1q may possess predictive value in aTAAD‐related POD. The present study aims to elucidate the potential association between C1q and POD development in patients with aTAAD, thereby offering valuable insights for the prediction and treatment of this postoperative complication.

## 2 Methods

### 2.1 Subject

This study is a secondary analysis of the data from a prospective observational study on neurological complications after type A aortic dissection surgery (registered at the Chinese Clinical Trial Registry, number: ChiCTR2200055980). Our study population encompassed 265 individuals who underwent TAAD surgical procedures at Nanjing Drum Tower Hospital from January 2022 to December 2022. Ethical clearance for this study was granted by the Medical Ethics Committee of Nanjing Drum Tower Hospital, the Affiliated Hospital of Nanjing University Medical School (IRB 2022‐034‐01), and it adhered to the ethical guidelines outlined in the Declaration of Helsinki for medical research involving human subjects. Prior to their enrollment, all patients provided written informed consent (The translation of the informed consent form template is in Supplementary File 1).

The inclusion criteria for participation in the study were delineated as follows: (1) patients aged 18 years or older; (2) a confirmed diagnosis of aTAAD with scheduled open thoracotomy aortic surgery; and (3) provision of signed informed consent. Conversely, the exclusion criteria encompassed: (1) a prior history of neurological or psychiatric disorders; (2) a history of severe cerebral infarction; (3) pregnancy; (4) the presence of malignant tumors; (5) patients in a terminally ill state with an anticipated lifespan of less than 24 h; (6) inability to undergo delirium assessment due to preoperative or postoperative coma, stroke, or other impediments; (7) insufficient or hemolyzed blood samples, or an absence of blood sample collection; and (8) any other conditions deemed ineligible for inclusion.

### 2.2 Experimental Desgin

In our study, blood samples of 3.5 mL were collected utilizing BD Vacutainer Collection Tubes (sourced from Melbourne, Australia), which were pre‐coated with spray‐coated silica and a polymer gel facilitating serum separation. Following collection, the tubes were promptly placed on ice for preservation. Subsequent to centrifuging the samples at 3000 revolutions per minute (rpm) for a duration of 10 min at a temperature of 4°C in a refrigerated centrifuge, the resultant serum was dispensed into 1.5 mL Eppendorf tubes (manufactured in Shanghai, China) and stored at a temperature of −80°C until further analysis could be conducted. The presence of serum complement component 1q (C1q) was quantified using a human C1q‐specific enzyme‐linked immunosorbent assay (ELISA) kit provided by Thermo Fisher Scientific (BMS2099TWO).

The specific time points for blood collection were as follows: (1) Preoperative (Pre), which occurred immediately upon patient admission; (2) Postoperative (Post), involving fasting blood samples collected at 6:00 a.m. on the first day subsequent to the surgical procedure; and (3) Awake (Final), where patients exhibiting delirium underwent blood sampling at 6:00 a.m. on the day following their awakening.

### 2.3 Measurements

Patients underwent emergency surgical interventions and perioperative management in accordance with our institutional protocols. Within the operating theater, standard cardiorespiratory monitoring was established to ensure patient safety. Anesthetic depth was meticulously assessed in all patients utilizing bispectral index (BIS) monitoring and quantitative electroencephalography (EEG). All patients were subjected to cryogenic circulatory arrest surgery, a complex procedure requiring precise anesthetic management.

Delirium was evaluated using the confusion assessment method (CAM) or its ICU‐specific variant, CAM‐ICU, whenever the Richmond Agitation‐Sedation Score (RASS) was ≥ −2. This assessment was conducted twice daily at 0800 and 2000 h for the initial three postoperative days, followed by once daily at 0800 h for the subsequent 4 days, encompassing both ICU and general ward settings. Patients who fulfilled the CAM or CAM‐ICU criteria during any of the postoperative screenings were classified as having delirium. The CAM and CAM‐ICU instruments evaluate delirium based on four core clinical features: (a) an acute onset of cognitive fluctuations, (b) inattention, (c) disorganized thinking, and (d) altered level of consciousness. A diagnosis of delirium was confirmed based on the presence of features (a) and (b), coupled with either (c) or (d). Baseline assessments for delirium were conducted prior to surgery, ensuring a comprehensive comparison. All assessments were performed by well‐trained and experienced investigators, ensuring the reliability and validity of the findings.

A comprehensive dataset was recorded for all patients, encompassing demographic characteristics such as age, sex, body mass index (BMI), and comorbidities (including hypertension, diabetes, smoking history, and alcoholism). Surgical details were meticulously documented, including the specifics of aortic root procedures, arch surgeries, any combined cardiac surgeries, duration of surgery, cardiopulmonary bypass (CPB) time, aortic cross‐clamp (ACC) time, deep hypothermic circulatory arrest (DHCA) time, and the lowest bladder temperature achieved. Preoperative (immediately following ICU admission) and postoperative (first day after surgery) laboratory tests were also recorded, encompassing a range of biomarkers including total bilirubin, albumin, glucose, creatinine, sodium (Na^+^), chloride (Cl^−^), white blood cell count (WBC), hemoglobin (Hb), and platelet count. The selection of these biomarkers was based on previous research and the specific clinical context of our institution, hypothesizing their potential relevance to the incidence of POD. Regarding surgical procedures, the stent treatment in aortic arch replacement referred specifically to the implantation of stents in both the whole arch and the descending aorta, whereas the descending aorta stent implantation involved the placement of stents solely within the descending aorta.

### 2.4 Statistical Analysis

All data were represented utilizing *n* (%) for categorical variables and mean ± standard deviation for continuous variables exhibiting normal distribution. For variables demonstrating non‐normal distribution, the median (interquartile range) was employed. The Kolmogorov–Smirnov test served as the method to ascertain the normality of the distribution. For variables adhering to normal distribution, either analysis of variance (ANOVA) or Student's *t*‐test was utilized. Categorical variables were appropriately analyzed using Fisher's exact test. Table 1, detailing the basic statistics, was generated utilizing the R package “tableone” (version 0.13.0). The predictive accuracy was assessed via the receiver operating characteristic (ROC) curve and the area under the ROC curve (AUC) using the “pROC” package (version 1.18.0). Statistical significance was established when *p* values were less than 0.05. To assess the representativeness of the included sample, we compared the baseline characteristics between the included patients and the total cohort using Student's *t*‐test for continuous variables and Pearson's chi‐square test for categorical variables. Results are presented in Supplementary Table S1. To evaluate the dose–response relationship, patients were categorized into quartiles (Q1–Q4) based on preoperative C1q levels. Multivariable logistic regression adjusted for cerebral perfusion was used to calculate ORs and 95% CIs for each quartile, with Q1 as the reference. The P for trend was calculated by treating quartile categories as a continuous ordinal variable in the same model.

**Table 1:** Baseline characteristics of patients in the non‐delirium and delirium groups.

	Non‐delirium (*n* = 30)	Delirium (*n* = 37)	*p*
Gender (male), *n* (%)	26 (86.7)	25 (67.6)	0.125
Age, years (mean ± SD)	50.40 ± 13.96	57.14 ± 15.30	0.067
Height, m (mean ± SD)	1.73 ± 0.07	1.67 ± 0.08	**0.001**
Weight, kg (mean ± SD)	79.87 ± 14.99	77.22 ± 14.14	0.460
BMI, kg/m^2^ (mean ± SD)	26.62 ± 4.24	26.70 ± 3.92	0.936
Smoke, *n* (%)	10 (33.3)	13 (35.1)	0.999
Alcohol, *n* (%)	8 (26.7)	7 (18.9)	0.644
Digestive diseases, *n* (%)	0	3 (8.1)	0.316
Peripheral vascular disease, *n* (%)	3 (10.0)	0 (0.0)	0.169
Nervous system disease, *n* (%)	1 (3.3)	7 (18.9)	0.115
Arrhythmology, *n* (%)	2 (6.7)	5 (13.5)	0.610
Myocardial infarction, *n* (%)	1 (3.3)	3 (8.1)	0.462
Hypertension, *n* (%)	25 (83.3)	32 (86.5)	0.988
Respiratory disease, *n* (%)	3 (10.0)	4 (10.8)	0.999
Diabete, *n* (%)	1 (3.3)	1 (2.7)	0.999
Renal insufficiency, *n* (%)	2 (6.7)	1 (2.7)	0.852
Consciousness, *n* (%)	1 (3.3)	3 (8.1)	0.763
Heart rate, bpm (mean ± SD)	81.06 (15.11)	78.89 (14.15)	0.547
Systolic pressure, mmHg (mean ± SD)	129.90 (25.16)	132.05 (23.72)	0.720
Diastolic pressure, mmHg (mean ± SD)	69.17 (15.22)	74.24 (16.66)	0.202
Mean blood pressure, mmHg (mean ± SD)	89.50 (16.31)	97.58 (19.09)	0.071
Surgery information			
Aortic root replacement, *n* (%)	5 (16.7)	6 (16.2)	0.999
Arch treatment, *n* (%)			
Replacement	8 (26.7)	16 (43.2)	0.362
Stent	7 (23.3)	6 (16.2)	
Descending aorta stent, *n* (%)	10 (33.3)	17 (45.9)	0.426
CPB, min (median [IQR])	181.00 [163.00–204.25]	189.00 [168.00–228.00]	0.200
ACC, min (median [IQR])	134.50 [115.75–151.28]	146.00 [118.00–189.00]	0.233
Cerebral perfusion, *n* (%)			
Anterograde	28 (93.3)	24 (64.9)	**0.013**
Retrograde	2 (6.7)	13 (35.1)	
DHCA time, min (median [IQR])	23.50 [20.00–29.00]	26.00 [22.00–30.00]	0.258
Lowest bladder temperature, °C (median [IQR])	24.00 [24.00–25.00]	24.00 [23.00–25.00]	0.553
Hospital stay time, days (median [IQR])	15.50 [13.00–20.75]	18.00 [15.00–27.00]	0.077
ICU stay time, hours (median [IQR])	98.75 [61.00–147.25]	182.00 [122.00–268.00]	**< 0.001**
Mechanical ventilation time, days (median [IQR])	0.80 [0.32–2.38]	2.30 [1.50–5.00]	**0.001**
Cerebral infarction, *n* (%)	3 (10.0)	8 (21.6)	0.344
Atrial fibrillation, *n* (%)	3 (10.0)	5 (13.5)	0.950
Pulmonary infection, *n* (%)	17 (56.7)	36 (97.3)	**< 0.001**
Hemiplegia, *n* (%)	0	3 (8.1)	0.316
Alimentary tract hemorrhage (%)	2 (6.7)	2 (5.4)	0.999
Acute kidney injury (%)	12 (40.0)	16 (43.2)	0.985
Hemiplegia (%)	0	3 (8.1)	0.316
Preoperative laboratory examination			
C1q Pre, ng/mL (median [IQR])	9.31 [6.75–10.38]	12.72 [9.95–15.85]	**< 0.001**
C1q Post, ng/mL (median [IQR])	6.60 [5.51–10.67]	9.97 [8.60–13.60]	**0.006**
C1q Final, ng/mL (median [IQR])	—	10.09 [7.74–11.05]	—
WBC, 109/L (median [IQR])	11.55 [8.83–14.33]	10.50 [8.20–13.10]	0.431
Neutrophil percentage, % (median [IQR])	86.90 [81.35–89.97]	86.90 [83.90–89.80]	0.650
Lymphocytes, 10^9^/L (median [IQR])	7.80 [4.95–10.38]	7.00 [5.40–10.30]	0.925
Monocytes, 10^9^/L (median [IQR])	5.20 [3.65–6.85]	5.70 [4.20–7.10]	0.553
Eosinophils, 10^9^/L (median [IQR])	0.00 [0.00–0.10]	0.10 [0.00–0.10]	0.320
Basophilic, 10^9^/L (median [IQR])	0.10 [0.10–0.10]	0.10 [0.00–0.20]	0.995
Hematocrit, % (median [IQR])	40.80 [33.38–43.15]	36.50 [31.50–40.40]	0.127
Hemoglobin, g/L (median [IQR])	124.50 [104.75–144.25]	121.00 [102.00–134.00]	0.226
Platelets, 10^9^/L (median [IQR])	160.00 [100.25–209.75]	129.00 [96.00–167.00]	0.133
Total bile acid, μmol/L (median [IQR])	20.65 [12.15–32.60]	15.40 [9.00–23.20]	0.100
Total protein, g/L (median [IQR])	63.25 [57.92–68.65]	61.60 [57.90–66.00]	0.512
Albumin, g/L (median [IQR])	37.80 [33.85–41.27]	38.20 [35.40–40.90]	0.955
Globin, g/L (median [IQR])	24.10 [21.00–26.92]	23.70 [20.30–26.00]	0.351
Glucose, mmol/L (median [IQR])	6.81 [5.56–7.98]	7.14 [6.40–9.08]	0.145
BUN, mg/dL (mean ± SD)	7.31 (3.13)	8.15 (2.48)	0.430
Creatinine, μmol/L (median [IQR])	76.45 [63.82–101.70]	92.00 [54.50–111.00]	0.990
Ttriglyceride, mmol/L (median [IQR])	1.23 [0.69–2.00]	0.67 [0.52–1.76]	0.085
Cholesterol, mmol/L (median [IQR])	3.34 [2.43–4.38]	3.43 [2.32–4.34]	0.747
HDL, mmol/L (median [IQR])	0.73 [0.71–1.29]	0.97 [0.73–1.40]	0.089
LDL, mmol/L (median [IQR])	2.20 [1.37–2.92]	2.15 [1.35–3.18]	0.767
APOA, g/L (median [IQR])	0.97 [0.78–1.17]	0.85 [0.70–1.03]	0.177
APOB, g/L (median [IQR])	0.70 [0.48–0.91]	0.64 [0.42–0.84]	0.397
Ca, mmol/L (median [IQR])	2.12 [2.04–2.34]	2.17 [2.04–2.26]	0.700
P, mmol/L (median [IQR])	1.08 [0.70–1.22]	1.05 [0.79–1.30]	0.686
K, mmol/L (median [IQR])	3.88 [3.73–4.35]	3.80 [3.40–4.26]	0.248
Na, mmol/L (median [IQR])	141.10 [138.10–145.50]	140.40 [136.80–143.60]	0.298
Cl, mmol/L (median [IQR])	105.45 [104.40–110.15]	103.80 [100.50–107.10]	0.092
C‐reactive protein, mg/L (median [IQR])	4.80 [2.42–27.50]	15.90 [2.50–38.00]	0.303

*Note:* Bold values are statistical significance (*p* < 0.05).

Abbreviations: APOA, apolipoprotein A; BMI, body mass index; BUN, blood urea nitrogen; Ca, serum calcium; Cl, serum chloride; DHCA, deep hypothermic circulatory arrest; HDL, high‐density lipoprotein; IQR, interquartile range; K, serum potassium; Na, serum sodium; P, serum phosphorus; SD, standard deviation; WBC, white blood cell.

## 3 Results

### 3.1 Patients

**Figure 1:**
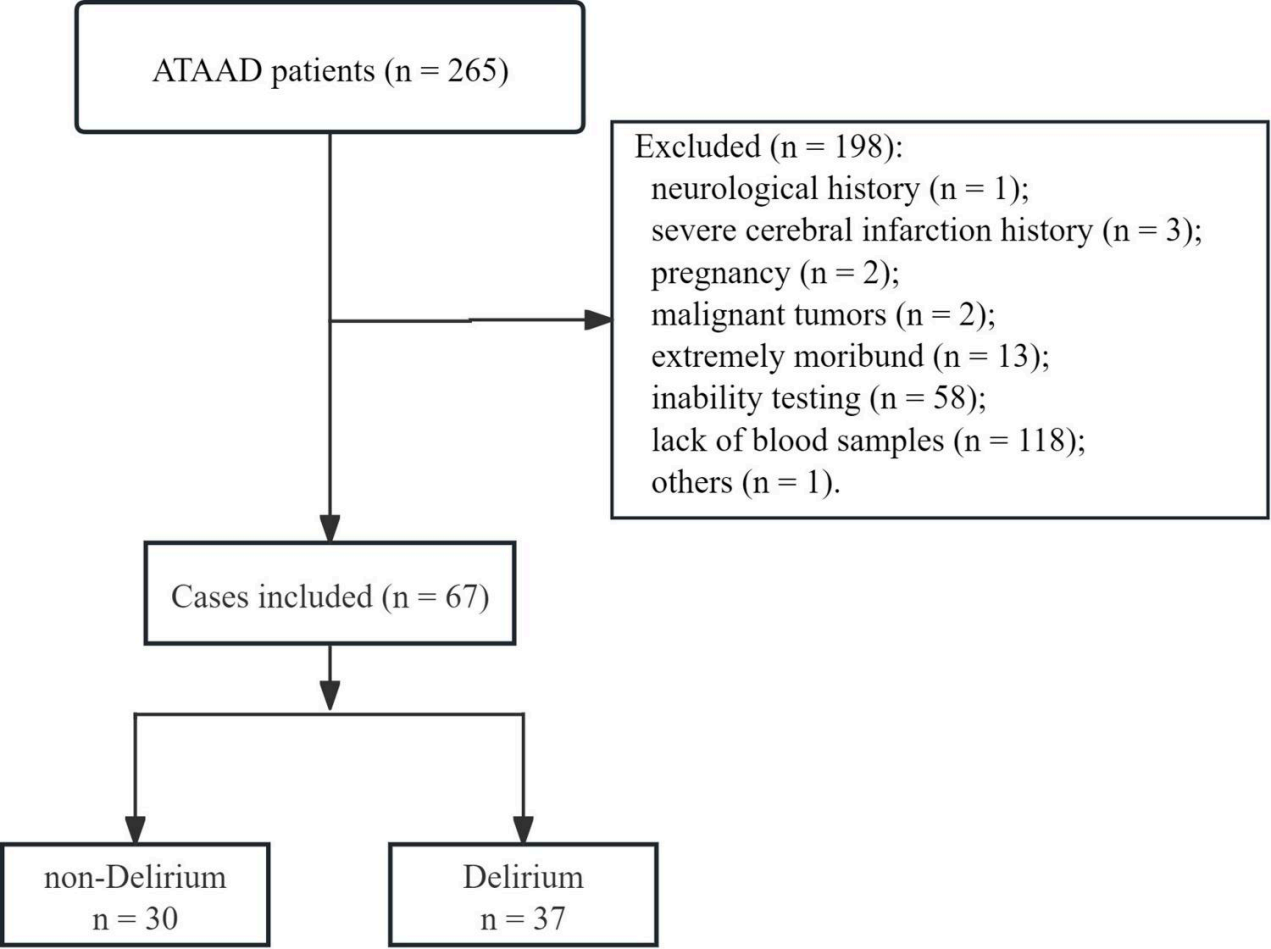
A flow chart of exclusion criteria is included in this study.

From January 2022 to December 2022, a total of 265 patients scheduled for aTAAD underwent screening. Based on meticulously established inclusion and exclusion criteria, a cohort of 67 patients was enrolled in this study (Figure 1). Comprehensive baseline demographics, surgical details, and laboratory findings were meticulously collected and documented for these patients (Table 1, Supplementary Table S2). Subsequently, the patients were stratified into two distinct groups: the delirium group (*n* = 37) and the non‐delirium group (*n* = 30), utilizing their Richmond agitation‐sedation scale (RASS) and confusion assessment method for the intensive care unit (CAM‐ICU) scores assessed on the first postoperative day as the basis for classification. A comparative analysis revealed that patients in the delirium group exhibited significantly prolonged durations of mechanical ventilation (*p* = 0.001) and intensive care unit (ICU) length of stay (*p* < 0.001) compared to those in the non‐delirium group.

### 3.2 Surum C1q Level

**Figure 2:**
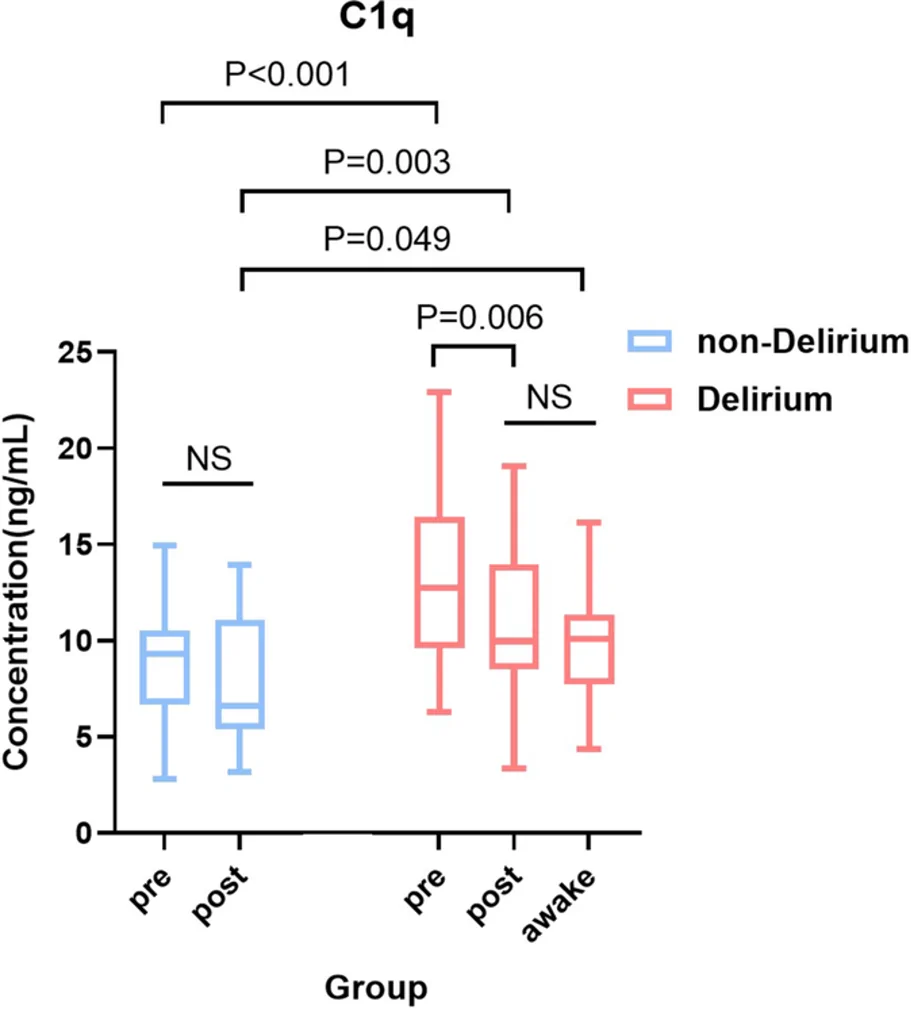
Serum c1q levels were detected in two groups at different time points. Pre, preoperative time point; post, the first day after surgery; awake, the next day after delirious patients assessed as awake.

We collected the serum of patients with aTAAD at two time points, before surgery and on the first day after surgery. Patients in the delirium group had an additional blood collection the next day after being assessed as awake. The preoperative C1q level in the delirium group was significantly higher than that in the non‐delirium group (12.72 [9.95–15.85] vs. 9.31 [6.75–10.38], *p* < 0.001, Table 1). Although there was a statistically significant decrease in serum C1q on the first day after surgery in patients with delirium (*p* = 0.006), there was no statistically significant difference in patients with non‐delirium (Figure 2). The level of serum C1q in the delirium group was still higher than that in the non‐delirium group on the first day after operation (*p* = 0.003). In addition, the serum C1q level of patients in the delirium group was still different from that in the non‐delirium group when they were taken again after waking up (*p* = 0.049).

### 3.3 Prediction Performance

Univariate logistic analysis was used to evaluate the predictive efficacy of serum C1q in POD after aortic dissection. The level of serum C1q before surgery (OR = 1.379, 95% CI = 1.176–1.683, *p*< 0.001) and the first day after surgery (OR = 1.236, 95% CI = 1.071–1.456, *p* = 0.006) can predict the occurrence of delirium well, and the preoperative C1q index performance is better (Table 2). Further multivariate logistic analysis showed that serum C1q and cerebral perfusion were more effective in predicting POD after aortic dissection, and patients with retrograde cerebral perfusion were more likely to have POD (Table 3).

**Table 2:** Univariate analyses of factors associated with POD.

Variables	OR	2.50%	97.50%	*B*	Wald	*p*
C1q Pre	1.379	1.176	1.683	0.321	12.605	**0.000**
C1q Post	1.236	1.071	1.456	0.211	7.429	**0.006**
Gender	3.120	0.945	12.352	1.138	3.144	0.076
Age	1.032	0.998	1.070	0.032	3.260	0.071
Height	0.000	0.000	0.014	−11.279	8.521	**0.004**
Nervous system disease	6.767	1.107	130.633	1.912	3.020	0.082
Cerebral perfusion	7.583	1.854	51.676	2.026	6.273	**0.012**
Glucose Pre	1.248	0.992	1.649	0.221	2.973	0.085
Platelet Pre	0.993	0.985	1.001	−0.007	2.790	0.095
Dexmedetomidine Pre	2.350	NA	3.014	−17.566	0.000	0.991
CPB	1.002	0.995	1.011	0.000	0.268	0.604
ACC	1.005	0.995	1.017	0.005	0.906	0.341
DHCA	1.001	0.952	1.056	0.001	0.003	0.954

*Note:* Bold values are statistical significance (*p* < 0.05).

Abbreviations: ACC, aortic cross‐clamp; CPB, cardiopulmonary bypass; DHCA, deep hypothermic circulatory arrest; OR, odds ratio.

**Table 3:** Multivariable logistic regression associated with POD.

Variables	OR	2.50%	97.50%	B	Wald	*p*
C1q Pre	1.457	1.212	1.853	0.377	12.436	**0.000**
Cerebral perfusion	12.792	2.465	106.075	2.549	7.517	**0.006**

*Note:* Initial candidate variables entered into the full model included C1q Pre, Cerebral perfusion, Age, Gender, Height, Nervous system disease, Platelet Pre, and Glucose Pre. Variable selection was performed using LASSO regression with 10‐fold cross‐validation (λ.1se), followed by clinical and statistical refinement. Height was retained by LASSO but excluded from the final model due to non‐significance in the adjusted analysis (*p* = 0.291) and lack of biological plausibility. ORs are adjusted for the other variable in the model. Bold values are statistical significance (*p* < 0.05).

Abbreviation: OR, odds ratio.

**Figure 3:**
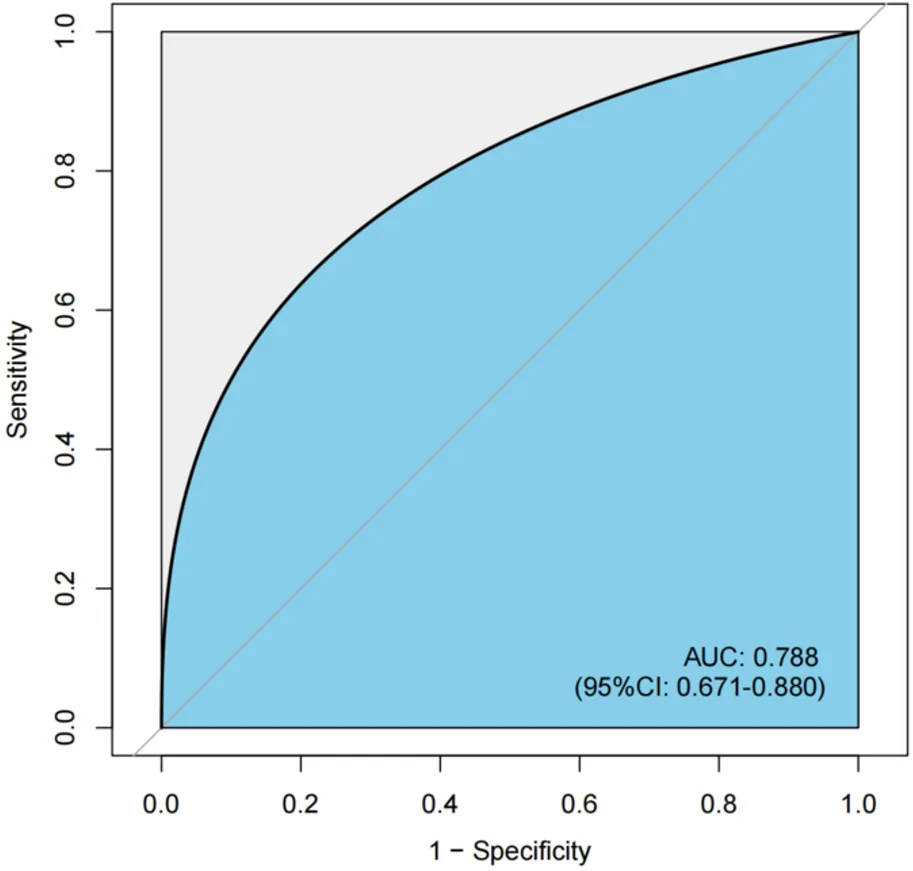
ROC curve of preoperative serum c1q predicting POD. AUC, area under the curve; CI, confidence interval; ROC, receiver operating characteristic.

The ROC curve of preoperative complement C1q as a predictor of POD after dissection has good performance (AUC = 0.788, 95% CI = 0.671–0.880, *p* < 0.001, sensitivity 65.4%, specificity 78.4%; Figure 3). We also calculated the correlation between complement C1q and some indicators (Figure S1).

### 3.4 Dose–Response Relationship Between C1q and POD

**Figure 4:**
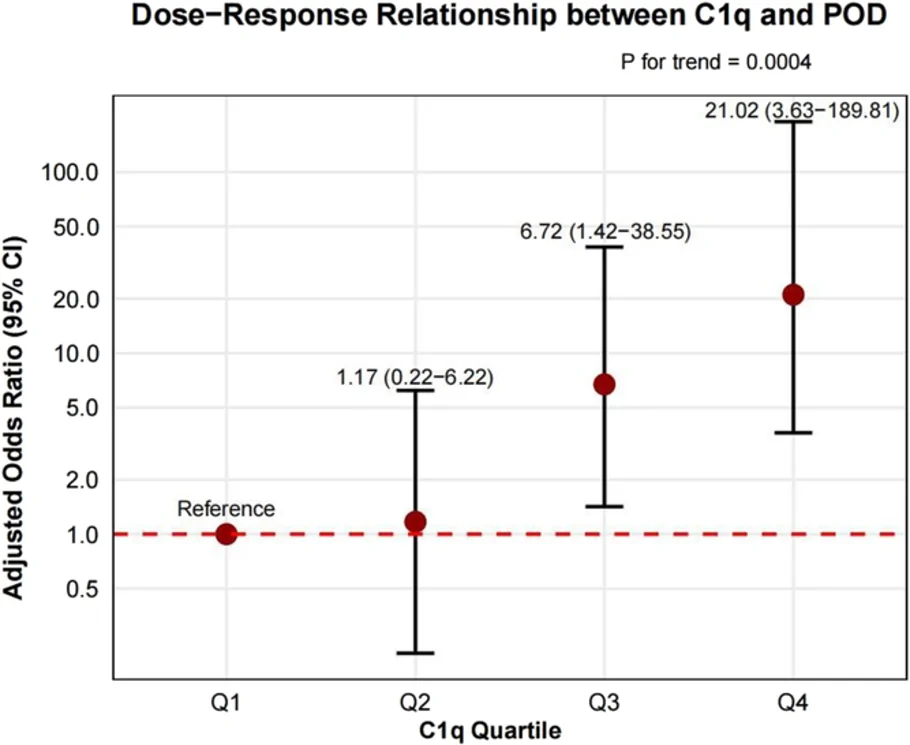
Dose–response relationship between preoperative C1q quartiles and risk of postoperative delirium.

To evaluate whether there is a dose–response relationship between preoperative C1q levels and POD, patients were divided into quartiles (Q1–Q4) based on C1q concentrations. Using the lowest quartile (Q1) as the reference, the adjusted ORs for Q2, Q3, and Q4 were 1.17 (95% CI: 0.22–6.22, *p* = 0.847), 6.72 (95% CI: 1.42–38.55, *p* = 0.022), and 21.02 (95% CI: 3.63–189.81, *p* = 0.002), respectively. A significant linear trend was observed across increasing C1q quartiles (*p* for trend = 0.0004), indicating a robust dose–response gradient between C1q level and POD risk (Figure 4).

## 4 Discussion

This study found that serum complement C1q in patients with delirium after TAAD was significantly higher than that in patients without delirium, and this difference persisted even after delirium patients regained consciousness. However, the mechanism by which complement C1q levels interact with delirium in patients with TAAD remains unclear.

In our study, the rate of POD in patients with aTAAD was about 55.2%, aligning with previous research findings [15]. Extensive literature exists on POD, with risk factors typically encompassing advanced age, preoperative hypohemoglobinemia, body mass index, preoperative renal insufficiency, history of cardiac surgery, duration of mechanical ventilation, and hypoxemia, among others [16]. In clinical practice, identifying high‐risk patients preoperatively is crucial for adopting streamlined surgical approaches or adequate cerebral perfusion strategies. Consequently, the pursuit of novel biomarkers with heightened specificity and precision is vital in the study of POD following TAAD [17, 18].

When comparing the predictive capacity of novel biomarkers for POD following surgery, preoperative serum C1q exhibited significant differences. Prior studies have implicated that delirium might be associated with systemic inflammatory mediators traversing the blood–brain barrier to activate microglial cells in the brain, thereby inducing neuroinflammation [19, 20]. In this study, there was a trend but no statistical difference in preoperative CRP between delirium and non‐delirium groups. This suggests that there may be a neuroinflammatory rather than systemic inflammatory response affecting the way. The role of serum C1q in cardiovascular and cerebrovascular diseases has been studied. Since C1q induces proliferation of vascular smooth muscle cells [21], elevated C1q levels have been shown to be a risk factor for atherosclerosis and arteriolar vasculopathy [22, 23].

The above studies suggest that C1q levels are elevated in patients with cardiovascular disease, but it is unclear whether this elevation in patients with post‐dissection delirium is due to the effects of dissection or to primary abnormalities in the brain. Recent studies also suggest that reducing C1q levels in sepsis‐associated encephalopathy (SAE) can prevent neuronal damage and improve neurocognitive outcomes [14]. The study used the oral C1q blocker PLX5622, which significantly reduced C1q levels in the central nervous system and is a promising drug candidate.

Our study demonstrates that elevated preoperative C1q levels are associated with a significantly increased risk of POD in patients with aTAAD, with a robust dose–response relationship (*p* for trend = 0.0004). These findings further support the potential role of complement pathway activation in the pathogenesis of perioperative neurocognitive disorders. Notably, this observation is not an isolated finding. Our group has recently reported that serum NPTX2—a marker reflecting synaptic plasticity and neuronal integrity—is also a promising predictive biomarker for POD in aTAAD patients, with an area under the curve of 0.895 for postoperative NPTX2 [18]. While NPTX2 primarily reflects neuronal vulnerability and synaptic dysfunction, C1q represents immune‐inflammatory activation via the complement cascade. Together, these two biomarkers provide complementary insights into the complex pathophysiology of POD, capturing both the “vulnerability” (neuronal/synaptic) and “trigger”(inflammatory/immune) dimensions of the disease [24].

Based on these findings, we propose the following potential measures. First, integrating C1q and NPTX2 measurements into preoperative assessment protocols could facilitate more comprehensive and multidimensional risk stratification, allowing identification of high‐risk patients who may benefit from enhanced perioperative management. Second, for patients identified as high‐risk by these biomarkers, intensified postoperative surveillance—including more frequent cognitive assessments (e.g., CAM‐ICU multiple times daily)—should be considered to enable early detection and timely intervention. Third, the convergence of evidence on complement activation (C1q) and synaptic dysfunction (NPTX2) suggests that modulating perioperative neuroinflammation or preserving synaptic integrity may represent promising therapeutic strategies for POD prevention. Complement pathway profiling may offer a novel approach for risk stratification and targeted therapeutic intervention in perioperative neurocognitive disorders. Agents targeting the complement cascade or those with neuroprotective properties warrant further investigation in prospective interventional studies.

Height was not retained in the final multivariable model for several reasons. First, its effect was highly sensitive to the unit of measurement—when expressed in meters (range: 1.60–1.85 m), the odds ratio was extremely close to zero and visually misleading; when converted to centimeters, the association became non‐significant (*p* = 0.291). Second, height lacked direct biological plausibility as a predictor of POD, and no established mechanism links adult height per se to postoperative neurocognitive outcomes [25]. However, we acknowledge that height may serve as a proxy indicator of physiological reserve, nutritional status, or frailty [26]—factors that are well‐documented to influence postoperative recovery and susceptibility to delirium [27, 28]. In this context, the apparent association between height and POD in univariate analysis may have been largely mediated through these intermediate factors. Indeed, frailty rather than height per se has been shown to be a stronger and more direct predictor of adverse postoperative outcomes [29]. A recent systematic review and meta‐analysis demonstrated that preoperative physical frailty is significantly associated with an increased risk of POD in older surgical patients (pooled OR: 2.47, 95% CI: 2.04–2.99) [27]. Had we adjusted for frailty indices or comprehensive nutritional scores, the effect of height might have been further attenuated. Therefore, while height was not retained as an independent predictor in our final model, its potential role as a marker of underlying vulnerability should not be overlooked. Future studies incorporating validated frailty assessments are warranted to disentangle the complex relationships among height, nutritional reserve, and POD risk [28].

However, several limitations must be acknowledged in this study. Given the sample size, the findings may not be generalizable to a broader population. The original study lacked comprehensive blood collection protocols, resulting in the exclusion of numerous patients due to inaccessible blood samples, potentially introducing selection bias. Ethical constraints precluded the investigation of the correlation between serum and cerebrospinal fluid C1q levels. We anticipate future research with a larger sample size to address these limitations.

## 5 What This Study Adds

The novelty of our study is threefold. First, this is a study to demonstrate that preoperative C1q levels are significantly elevated in patients who subsequently develop POD, establishing a link between complement pathway activation and POD in patients with aTAAD. Importantly, this difference is detectable before surgery, suggesting that C1q serves as a pre‐existing susceptibility marker rather than a mere acute‐phase response to surgical stress.

Second, we identified a robust dose–response relationship between C1q levels and POD risk (*p* for trend = 0.0004), with patients in the highest C1q quartile having a 21‐fold increased risk compared with those in the lowest quartile. This gradient effect supports C1q as a graded risk indicator for preoperative stratification and enhances its clinical utility.

Third, integrated with our recent work on NPTX2—a marker of synaptic plasticity and neuronal integrity—our findings suggest that complement‐mediated neuroinflammation and synaptic dysfunction may represent converging pathways in POD pathogenesis [18]. While NPTX2 reflects neuronal vulnerability, C1q represents immune‐inflammatory activation via the complement cascade. Together, these biomarkers provide complementary insights into the complex pathophysiology of POD, and point to complement pathway activation as a potentially modifiable therapeutic target.

## 6 Conclusions

In our investigation, we conducted a prospective analysis of serum biomarkers specifically targeting C1q. The results revealed that preoperative C1q levels exhibit considerable promise as indicators of POD following surgical intervention for aTAAD. This discovery holds the potential to identify a novel target for therapeutic interventions aimed at preventing the occurrence of POD.

## Author Contributions

**Jianing Wang:** writing – review and editing. **Yi Jiang:** writing – review and editing, formal analysis, writing – original draft. **Shan Lu:** investigation, funding acquisition. **Wenxue Liu:** data curation, validation. **Yapeng Wang:** investigation. **Jiawei Zhu:** investigation. **Xiujuan Cai:** validation. **Yuhuan Tian:** investigation. **Yongqing Cheng:** methodology. **Yunxing Xue:** conceptualization, funding acquisition. **Dongjin Wang:** conceptualization.

## Ethics Statement

All the tested individuals provided informed consent to participate in the study. All samples were obtained and analyzed in accordance with the Medical Ethics Committee of Nanjing Drum Tower Hospital, the Affiliated Hospital of Nanjing University Medical School, China (IRB 2022‐034‐01). The study was carried out in accordance with the International Ethical Guidelines and the Declaration of Helsinki. Trial registered in the Chinese Clinical Trial Registry (http://www.chictr.org.cn/), number ChiCTR2200055980 (registered date of January 30, 2022). This trial was registered before the first participant was enrolled.

## Consent

Informed consent was obtained from all subjects involved in the study.

## Conflicts of Interest

The authors declare no conflicts of interest.

## Supporting information


**Figure S1:** Comparison of baseline characteristics between included patients and the overall cohort

**Table S1:** Baseline characteristics of patients in the non‐Delirium group and Delirium group
**Table S2:** Correlation analysis between serum C1q level and some indexes

Supporting File 1

Supporting File 2

Supporting File 3

Supporting File 4

## Data Availability

The data that support the findings of this study are available on request from the corresponding author. The data are not publicly available due to privacy or ethical restrictions. The data presented in this study are available on request from the corresponding author.
