# Supplementary material for: Serum C1q as a Novel Predictive Biomarker for Postoperative Delirium After Acute Type A Aortic Dissection
Source: Immun Inflamm Dis. 2026 Sep 8;14(9):e70500. doi: 10.1002/iid3.70500 (PMC13552218; doi:10.1002/iid3.70500)
Supplement: Supplementary file 1 [file IID3-14-e70500-s005.pdf]

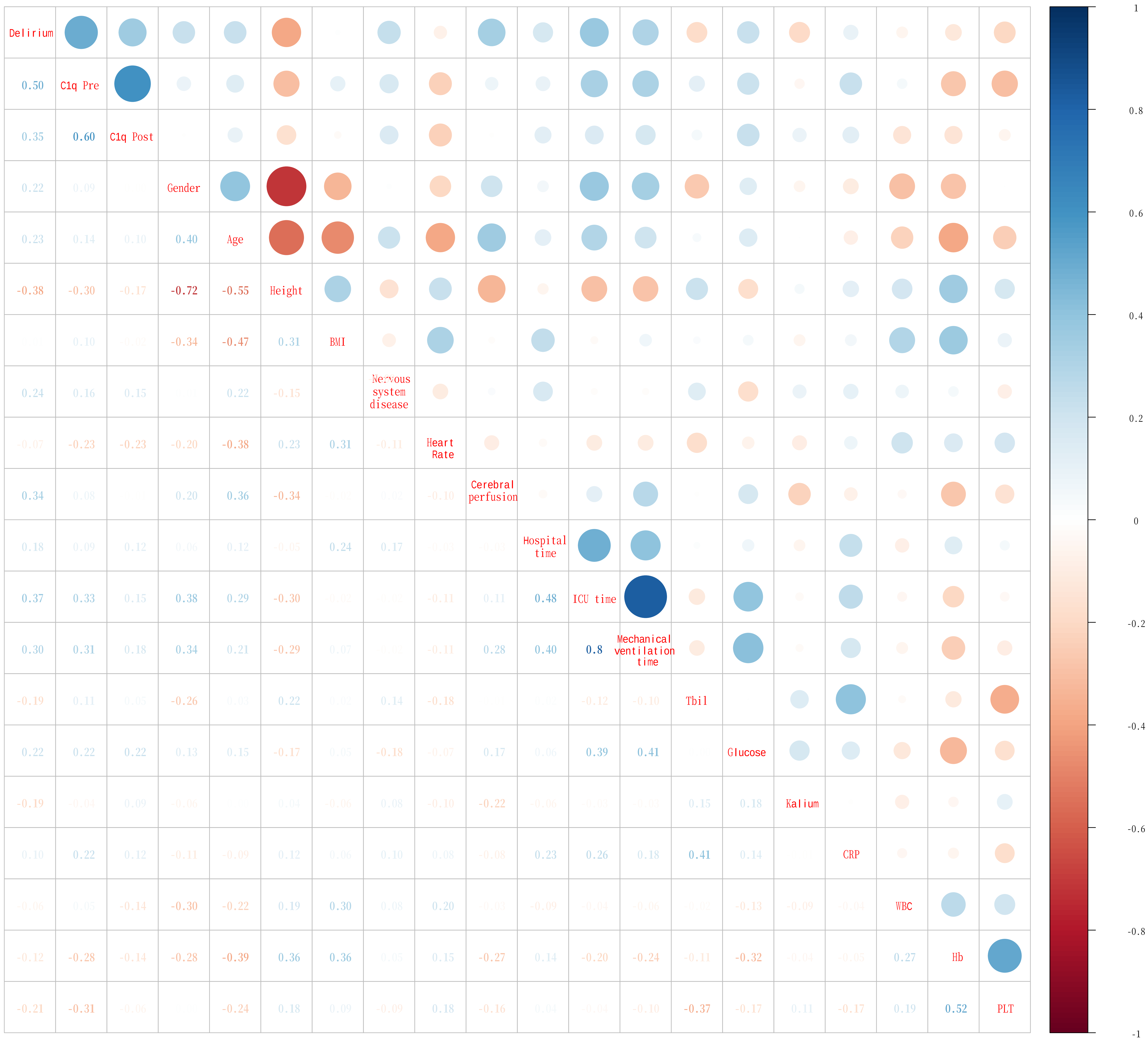
Delirium
C1q Pre
C1q Post
Gender
Age
Height
BMI
Nervous system disease
Heart Rate
Cerebral perfusion
Hospital time
ICU time
Mechanical ventilation time
Tbil
Glucose
Kalium
CRP
WBC
Hb
PLT
1
0.8
0.6
0.4
0.2
0
-0.2
-0.4
-0.6
-0.8
-1
