# Supplementary material for: Serum C1q as a Novel Predictive Biomarker for Postoperative Delirium After Acute Type A Aortic Dissection
Source: Immun Inflamm Dis. 2026 Sep 8;14(9):e70500. doi: 10.1002/iid3.70500 (PMC13552218; doi:10.1002/iid3.70500)
Supplement: Supplementary file 2 [file IID3-14-e70500-s002.docx]

Supplementary File 2

Supplementary Table S3. Full multivariable logistic regression model including all 8 candidate variables

| Variable | Adjusted OR | OR 95CI | P value |
| --- | --- | --- | --- |
| C1q Pre | 1.420 | 1.42 (1.119 – 1.803) | 0.004 |
| Cerebral perfusion | 14.033 | 14.033 (1.519 – 129.659) | 0.020 |
| Age | 0.971 | 0.971 (0.914 – 1.031) | 0.335 |
| Gender | 1.152 | 1.152 (0.107 – 12.376) | 0.907 |
| Height | 0.004 | 0.004 (0 – 4636.388) | 0.441 |
| Nervous system disease | 4.578 | 4.578 (0.391 – 53.651) | 0.226 |
| Platelet Pre | 1.002 | 1.002 (0.991 – 1.013) | 0.733 |
| Glucose Pre | 1.248 | 1.248 (0.906 – 1.719) | 0.175 |

ORs are adjusted for all other variables in the model. Gender and Nervous_system_disease were coded as binary variables (1 = presence of the characteristic). Height was measured in meters; the extremely wide confidence interval reflects unit sensitivity (see Results section for details).

Supplementary Table S4. Variables retained by LASSO regression at λ.1se

| Variable | Coefficient |
| --- | --- |
| (Intercept) | 1.697 |
| C1q Pre | 0.155 |
| Cerebral perfusion | 0.664 |
| Height | -1.981 |

LASSO regression was performed with 10-fold cross-validation. The optimal tuning parameter λ was selected using the one‑standard‑error rule (λ.1se). Variables with non‑zero coefficients at λ.1se are listed. All other candidate variables had coefficients shrunk to zero.

Figure S1A

Figure S1B

Figure S1. LASSO regression for variable selection. (A) Cross-validation curve showing binomial deviance as a function of –Log(λ). (B) Coefficient paths for the 8 candidate variables. Each colored line represents the trajectory of a variable’s coefficient as –Log(λ) increases (i.e., as the penalty λ decreases). Based on the cross-validation results, the optimal λ was selected, at which only three variables retained non-zero coefficients (C1q Pre, Cerebral perfusion, and Height) and were subsequently included in the final model.
