# Supplementary material for: Serum C1q as a Novel Predictive Biomarker for Postoperative Delirium After Acute Type A Aortic Dissection
Source: Immun Inflamm Dis. 2026 Sep 8;14(9):e70500. doi: 10.1002/iid3.70500 (PMC13552218; doi:10.1002/iid3.70500)
Supplement: Supplementary file 3 [file IID3-14-e70500-s004.docx]

Supplementary Material: Detailed Statistical Methods (STROBE Items 9, 10, 12c)

Item 9 – Bias (Potential sources of bias and efforts to address them)

To minimize potential bias, the following measures were implemented. First, selection bias was addressed by applying consecutive enrollment of eligible participants according to pre-specified inclusion and exclusion criteria. Second, to reduce information bias, all clinical data were collected prospectively using standardized case report forms, and laboratory measurements (including C1q levels) were performed by trained technicians blinded to patient outcomes. Third, to control for confounding bias, we adopted a multivariable logistic regression model adjusting for clinically relevant covariates identified a priori based on based on clinical knowledge and literature. Fourth, the potential for reverse causality was minimized by measuring the exposure (C1q) prior to the outcome (POD) in this prospective cohort design.

Item 10 – Study Size (How the study size was determined)

The sample size of the original study was calculated based on the anticipated overall incidence of the primary outcome (approximately 30%). Using the single-proportion estimation method with a two-sided significance level (α = 0.05), statistical power (1-β = 80%), and a margin of error of 10%, the required sample size was estimated to be 256 participants. The present study ultimately enrolled 265 eligible participants, meeting the pre-specified design requirement.

For the secondary analysis of the association between C1q and POD, we further evaluated the adequacy of the sample size using the Events Per Variable (EPV) criterion. With 37 positive outcome events and 3 independent variables in the multivariable logistic regression model, the EPV was calculated as 37/3 = 12.33, exceeding the recommended threshold of 10 proposed by Peduzzi et al. (1996), thereby confirming sufficient statistical power for the primary analysis.

Item 12c – Missing Data (How missing data were addressed)

The handling of missing data followed established guidelines for observational research . The overall exclusion rate was 74.7%, primarily attributable to blood sample quality issues (accounting for 59.6% of all excluded cases), with the remainder due to other predefined exclusion criteria. The high exclusion rate was due to objective factors in this emergency surgical setting (acute type A aortic dissection, aTAAD): some patients could not provide sufficient blood samples due to preoperative resuscitation efforts or limited venous access, while other samples were hemolyzed, making C1q measurement impossible.

To assess the potential impact of missing data on the validity of our findings, we compared baseline characteristics between the included and excluded groups. As shown in the main text (Table 1), there were no statistically significant differences between the two groups in terms of sex (P = 0.125), age (P = 0.067), weight (P = 0.460), body mass index (P = 0.936), smoking (P = 0.999), alcohol consumption (P = 0.644), and major comorbidities (all P > 0.05). This consistency suggests that missingness was unlikely to be systematically associated with patient characteristics.

Complete case analysis was performed for the multivariable logistic regression, as the proportion of missing data for the analytical variables was minimal after exclusions. Given that the high rate of missing data was driven by a specific, objectively verifiable cause (sample hemolysis or insufficient volume) rather than patient prognosis, we did not employ multiple imputation; the comparison of baseline characteristics between included and excluded groups further supported that the missing data were unlikely to introduce substantial selection bias .
