# Supplementary material for: Serum C1q as a Novel Predictive Biomarker for Postoperative Delirium After Acute Type A Aortic Dissection
Source: Immun Inflamm Dis. 2026 Sep 8;14(9):e70500. doi: 10.1002/iid3.70500 (PMC13552218; doi:10.1002/iid3.70500)
Supplement: Supplementary file 4 [file IID3-14-e70500-s006.docx]

**Informed consent form**

Dear Ladies/Men:

We are about to conduct a study on the predictive value of quantitative electroencephalography (qEEG) for brain function in patients with type A aortic dissection (aTAAD) (study title). You may meet the eligibility criteria for this study, so we invite you to participate. The sponsor of this study is the Department of Cardiothoracic Surgery at Nanjing University Medical School Affiliated Drum Tower Hospital, and the principal investigator is Associate Chief Physician Cheng Yongqing.

This informed consent form will introduce you to the purpose of the study, its procedures, the benefits it brings, the risks, inconveniences, or discomforts you may face, and the main points of the research. It also includes your right to withdraw from the study at any time. Please read this carefully before making a decision on whether to participate in the study. This informed consent form may contain terms or information that you do not understand; please consult your research doctor for clarification until you are satisfied. Before making a decision, you can take the unsigned informed consent form home to consider or discuss with your family, friends, or anyone else you choose. If you decide to join this study, please sign and date this informed consent form. Your signature will not deprive you of any legal rights. The original signed informed consent form will be kept by the researcher, while a copy will be retained by you.

**1. Research background**

Aortic dissection (Aortic Dissection, AD) is a critically severe cardiovascular disease with an extremely high mortality rate, with an incidence of about 2.78 per 100,000 person-years in China. Untreated acute Type A aortic dissection (acute Stanford type A Aortic Dissection, aTAAD) has a mortality rate that increases by 1% per hour over time, reaching as high as 50% within 48 hours. Conservative medical treatment is ineffective, with a mortality rate as high as 57%. With continuous advancements in medical technology, the 30-day mortality rate after aTAAD has decreased from 30% to 12.2%. However, due to aTAAD, which often presents acutely and is accompanied by poor organ perfusion, surgery requires deep hypothermic circulatory arrest and longer extracorporeal circulation times, leading to high postoperative complications. Among these, neurological injury is one of the most severe complications, significantly impacting patient outcomes. ATAAD often results from cerebral perfusion impairment secondary to endarterial involvement of the neck vessels, occlusion, thromboembolism, or hypotension. However, preoperative coma and stroke in aTAAD patients are independent predictors of late mortality due to their risk of inducing hemorrhagic infarction and poor long-term prognosis, making them contraindicated for surgery. Improving the prognosis of preoperative patients with neurological deficits remains a challenge in cardiac and vascular surgery. Additionally, studies have shown that the incidence of postoperative delirium in aTAAD patients is approximately 32%. 5 – 52.0%, mental disorders account for about 51.5%, and the incidence of post-traumatic stress disorder syndrome is approximately 32%. Its occurrence is closely related to increased mortality, higher medical costs, cognitive impairment, and decreased quality of life. Studies have shown that neurofunctional monitoring can effectively guide perioperative care and treatment in patients with neurological deficits. Currently, commonly used neurofunctional monitoring methods in clinical practice include intracranial pressure, cerebral oxygenation, somatosensory evoked potentials, and electroencephalography. Among these, quantitative EEG monitoring is particularly simple and easy to perform

Operations that can directly reflect neuronal activity and other advantages have been widely applied in clinical practice in recent years. Evidence shows that flat, burst suppression, or status epilepticus brain electrical activities are closely related to poor patient outcomes. Other studies indicate that reduced EEG amplitude, increased θ and δ waves, and decreased α waves are associated with postoperative delirium and cognitive impairment in patients. Based on the above evidence, we speculate that perioperative EEG monitoring may be beneficial for reducing mortality rates and

Neuropathy may have predictive effect, which provides scientific basis for early identification, timely intervention and timely evacuation.

**2. Purpose of the study**

To clarify the predictive effect of qEEG on brain function prognosis in aTAAD patients.

**3. Conditions for participation in the Institute**

This study is a single-center, observational study that continuously enrolled 210 patients who were admitted to our hospital for aTAAD within the specified time frame. Inclusion criteria: ① Patients primarily diagnosed with ATAAD; ② Patients expected to undergo open surgery due to ATAAD; ③ Participants can be of any gender and must be at least 18 years old; ④ Participants must have signed an informed consent form. Exclusion criteria: ① Patients with a history of neurological or psychiatric disorders, recent cerebral infarction within six months, or pregnancy; ② Patients with confirmed malignant tumors that significantly affect life expectancy; ③ Participants under 18 years old. The first enrolled participant underwent surgery on February 1, 2022, and the last case was included

The operation time of the subjects was February 01,2024. The study will follow up and provide health guidance to the subjects.

**4. Number of people studied**

About 210 subjects are planned to be enrolled in this trial.

**5. Is it necessary to participate in and complete this study?**

Whether you participate in this trial is entirely at your own volition. If you decide to participate, you will be asked to sign an informed consent form and will receive a copy of this informed consent form. If you participate in this study, you can still withdraw at any time without affecting your standard treatment.

**6. Research process**

The doctor will collect your demographic data, history of heart disease, cardiac function status, surgical information, intraoperative qEEG and related neurological imaging data (if any), postoperative qEEG, postoperative stroke, delirium, post-traumatic stress disorder, anxiety, depression, cognitive function, relevant laboratory indicators, mechanical ventilation time, tracheostomy, pulmonary infection, and other clinically relevant events through the hospital's electronic medical records. The doctor will also inquire about your post-aTAAD surgery recovery status at three months, six months, one year, and two years after discharge, including post-traumatic stress disorder, anxiety, depression, cognitive function, quality of life, disease rehabilitation, lifestyle habits, medication use, and any recurrence or hospitalization.

In this study, blood gas analysis waste samples of about 2ml were collected before and after operation. The waste blood was collected into the biochemical tube, and the serum was stored in the cardiothoracic surgery-80℃ refrigerator after centrifugation for immunomicroscopy Factor detection and other related studies. If there are still remaining samples, they will be destroyed. This study does not affect the normal diagnosis and treatment process of patients, patients can apply for termination or withdrawal at any time, without commercial conflict of interest.

Related costs: This study does not require any examination or treatment. You will not be paid for participating in this study, and you will not incur any additional expenses.

**7. Participate in the research and what you need to do to complete it**

◆ You only need to truthfully answer questions regarding your recovery from the ATAAD surgery, your lifestyle habits, medication use, and any recurrence or hospitalization after discharge. Please also agree that your follow-up information and clinical treatment data from the original hospitalization or clinic visits will be used for this medical study. If you have any other questions about your recovery, please provide them to the follow-up personnel as well.

**8. Alternative treatment options if this trial is not participated in**

You can choose not to participate in this study, which will have no adverse effects on you.

**9. Possible side effects, risks and discomforts associated with participation in the trial**

This study is an observational study and will not involve any medical intervention. The data collected from this study, including your general information, perioperative data, and in-hospital records, all come from the existing data in the hospital's case system. Follow-up data were obtained during subsequent follow-up processes. The medical center will remove identifiable information (such as name, address, phone number) from your clinical data before aggregating and analyzing these data. Therefore, it will not pose any additional risk to you.

**10. Possible benefits of participating in the trial**

The study follow-up process will help promote your personal health management after surgery, provide you with timely health education and medication guidance, and the results of the study may help identify potential adverse risks associated with ATAAD surgery to enable you and other subjects with similar conditions to obtain higher quality medical services in the future.

**11. Your rights**

Participation in the study is entirely voluntary. You can withdraw your informed consent at any time without giving a reason. If you decide to withdraw from the study (stop the study), you must inform your attending physician; your attending physician may ask and record relevant health conditions and reasons for withdrawal.

You may not fully understand the contents of this informed consent form. You can consult your research doctor about the study

If you have any questions during the process, you can also consult your research doctor at any time.

**12. Fees for participation in this study/experiment**

There are no additional expected costs for participation in this trial.

You will not be paid for taking part in this study.

**13. Privacy and confidentiality**

Any information and data obtained about you during the trial will be kept strictly confidential. Information that can identify your identity will not be disclosed to members outside the research team unless you give permission. All members of the research team and sponsors are required to keep your identity confidential. Your records will be stored in a locked file cabinet for researchers to access only. To ensure the study is conducted according to regulations, when necessary, government regulatory authorities, authorized monitors by the sponsor, or members of the ethics committee may review your information related to the trial at the research institution, but they will ensure that your information is not disclosed to any other party. Although the results of the study may be published, your identity will not be revealed in these publications. The data from this study will be stored at the Affiliated Nanjing Drum Tower Hospital of Nanjing University Medical School.

By signing this written informed consent, you indicate that you have agreed to the study physician collecting and processing your personal information ("study data") in this research, including: your date of birth, gender, race, and personal data on physical and mental health. Unless you withdraw your informed consent, it means your study data will remain available for use. If you withdraw your informed consent, the study physician and sponsor will no longer use your personal data, but any personal data shared before the withdrawal of informed consent can still be used.

The investigator will use the data for clinical studies. The sponsor may use the data for: conducting clinical studies, supporting applications for marketing authorization of investigational drugs and developing new drug products, diagnostics, or medical aids.

You have the right to request that your personal data be retained by your research physician and sponsor, and you also have the right to request that inaccuracies in your personal data be corrected; you have the right to withdraw your informed consent at any time. If you have such requests, please contact your research physician.

**14. Contact information**

◆ If you have further questions, please contact:

Name of doctor: address: contact number:

◆ If you have any questions related to the rights and interests of the subjects, please contact the Medical Ethics Committee of Nanjing Drum Tower Hospital affiliated to Nanjing University School of Medicine at 025-68182923

**Informed consent form signature page**

Informed consent statement of the subject:

• I have read this informed consent form, and have been provided with the background, purpose, procedures, risks and benefits of this trial. I have had sufficient time and opportunity to ask questions about this clinical trial, and have been satisfied with the answers.

• I understand that participation in this trial is voluntary.

• I allow the use and sharing of my medical information as described in the informed consent.

• I know that I can withdraw from this trial at any time without suffering any loss of benefit or other adverse consequences.

• I am willing to cooperate with the researchers for relevant examination or treatment.

• I know that my personal identity and privacy will be kept strictly confidential in this study.

• I was also informed who to contact if I had questions or wanted further information.

• I will receive a copy of this informed consent signed and dated.

Subject's signature (printing): Contact number:

Subject's signature (handwritten): date:

Signature of the guardian [if applicable] (print, please indicate the direct relationship with the subject):

contact number:

Guardian's signature (handwritten): date:

Statement of the investigator conducting an informed consent study:

I or my research team have fully explained and informed the subject of the background, purpose, procedures, risks, and benefits of this clinical trial, giving him/her sufficient time to read the informed consent form, discuss with others, and answer any questions related to the study; I have provided the subject with contact information in case of any issues; I have also informed the subject (or guardian) that he/she can withdraw from the study at any time during the study period without providing any reason.

Signature of the investigator (printing): Contact number:

Researcher signature (handwritten): date:

知情同意书 受试者须知

尊敬的女士/先生：

我们将要开展一项关于定量脑电监测（qEEG）对 A 型主动脉夹层（aTAAD）患者 脑功能预后预测价值的研究（研究题目），您可能符合该项研究的入组条件，因此，我 们邀请您参加该项研究，该研究的申办方是南京大学医学院附属鼓楼医院心胸外科，主 要研究者是程永庆副主任医师。

本份知情同意书将向您介绍该研究的目的、步骤、给您带来的益处、您要承担的风 险、不便或不适和研究主要事项，以及您有在任何时候退出研究的权利。请您仔细阅读 后慎重做出是否参加研究的决定。本份知情同意书可能包含一些您不理解的词语或信 息，请一定咨询您的研究医生，您将得到回答直到您满意。做决定前， 您可以将未签字 的知情同意书带回家考虑或与您的家人、朋友或任何您选择的对象商量。如果您决定参 加本研究，请您在这份知情同意书上签名和签署日期。您的签名不会使您丧失任何合法 权益，签字后的知情同意书原件将保留在研究者处，另一份副本由您自己留存。

**1. 研究背景**

主动脉夹层（Aortic Dissection, AD）是一种致死率极高的心血管病危急重症， 我国发病率约为 2.78 每 10 万人年。不经治疗的急性 A 型主动脉夹层（acute Stanford type A Aortic Dissection, aTAAD）死亡率随着时间推移，每一个小时死亡率增高 1%， 48 小时死亡率高达 50%,药物保守治疗效果不佳，死亡率高达 57%。随着医疗技术水平的 不断进步，aTAAD 术后 30 天死亡率从 30%下降到 12.2%。然而，由于 aTAAD 往往急性起 病、伴随脏器灌注不良， 手术需要深低温停循环、体外循环手术时间长等不良因素，导 致术后并发症高，其中神经系统损伤是最严重的并发症之一，严重影响患者预后。ATAAD 往往由于夹层内膜累及颈部血管受损、闭塞、血栓栓塞或低血压而继发的脑灌注不良。 但是，aTAAD 患者术前昏迷和卒中是晚期死亡率的独立预测因素，由于其有诱发出血性 脑梗死的风险和较差的长期预后，因此被认为是手术禁忌。改善术前合并神经功能障碍 患者的预后仍是心脏大血管外科的挑战。另有研究显示，aTAAD 术后谵妄的发生率约为 32.5 –52.0%，精神障碍约为 51.5%，创伤性应激障碍综合征的发病率约为 32%。其发生 与死亡率升高、医疗费用增加、认知功能损伤以及生活质量下降等密切相关。研究表明， 神经功能监测可有效指导神经功能损伤患者围术期的监护治疗。目前， 临床常用神经功 能监测方法有颅内压、脑氧、体感诱发电位、脑电等。其中， 定量脑电监测因其简便易

操作，可直接反应神经元活动等优点，近年来被广泛应用于临床实践中。证据表明， 脑 电活动表现为平坦、暴发抑制或癫痫持续状态等与患者预后不良密切相关。另有研究指 出，患者脑电振幅降低， θ 、 δ 脑电波增加， α 脑电波降低等与患者术后谵妄及认知 功能损伤相关。基于上述证据， 我们推测，围术期脑电检测对 aTAAD 患者术后死亡率及

神经功能损伤可能具有预测作用。为提前识别、及时干预、适时撤离等提供科学依据。

**2. 研究目的**

明确 qEEG 对 aTAAD 患者脑功能预后的预测作用。

**3. 参加研究所要满足的条件**

本研究为单中心、观察性研究，将连续入选在规定时间内在我院接受aTAAD 患者 210 例，入选标准：①ATAAD 为主要诊断入院的患者；②因 ATAAD 预计进行开放手术的 患者；③受试者男女不限，年龄区间为≥18 岁；④签署知情同意书。排除标准： ①既往 神经、精神病史、半年内脑梗、妊娠状态；②明确恶性肿瘤，严重影响预期寿命的患者 除外，③年龄<18 岁。首例入选受试者的手术时间为 2022 年 02 月 01 日，最后一例入选

受试者的手术时间为 2024 年 02 月 01 日。研究将对受试者进行随访及健康指导。

**4. 研究的人数**

本试验计划入选约 210 名受试者。

**5. 是否一定要参加并完成此项研究？**

您是否参加此项试验完全出于您的自愿，假如您决定参加，您将被要求签署知 情同意书并且会获得此知情同意书的副本。如果您参加了此项研究，您仍可以随时 要求退出，若您退出不会影响您的标准治疗。

**6. 研究过程**

医生会通过医院的电子病历收集您的人口学资料、心脏病史、心功能状态、手 术资料、术中 qEEG 神经相关影像学资料（如有）、术后 qEEG、术后卒中、谵妄、 创伤后应激障碍、焦虑、抑郁、认知功能、相关化验室指标及机械通气时间、气管 切开、肺部感染等临床相关事件等资料。并于术后三月、半年、一年、两年询问您 接受 aTAAD 手术出院后的创伤后应激障碍、焦虑、抑郁、认知功能、生活质量疾病 康复情况、生活习惯、用药情况和再次发病或住院等信息。

本研究将留取术前、术后 1 小时和术后连续 3 天的血气分析废弃血样本，约 2ml， 将废弃血收集至生化管，离心之后血清保存在心胸外科-80℃冰箱，进行免疫组学、

因子检测等相关研究。若仍有剩余样本，一律销毁。本研究不影响患者正常诊疗流 程，患者可随时申请终止或撤回，无商业利益冲突。

有关费用：本研究不需任何检查和治疗，您参加本项研究，我们不支付报酬， 同时也不会给您造成任何额外花费。

**7. 参加研究，需要您配合完成的事**

◆	您只需依照自身情况如实回答您接受 ATAAD 手术出院后的疾病康复情况、生 活习惯、用药情况和再次发病或住院等信息，并且同意您的随访信息及原住院/ 就诊期间的临床诊疗信息用于本次医学研究。如有其它关于疾病康复方面的问 题，可一并提供给随访人员。

**8. 如果不参加此项试验，其他可备选的治疗方案**

您可以选择不参加本项研究，这对您不会带来任何不良影响。

**9. 参加试验可能出现的副作用、风险以及不适**

本研究为观察性研究，不会对您进行任何医疗干预。本研究获得的资料包括您的 一般资料、围术期资料和院内资料等均来自医院病例系统中现有资料， 随访资料由后 期随访过程获得。医疗中心将在去除您临床资料中的可识别信息（如姓名、住址、电 话等） 之后，再对这些数据进行汇总以及分析。因此， 不会给您造成任何额外风险。

**10. 参加试验可能的获益**

研究随访过程有助于促进术后您个人的健康管理，给予您及时的健康宣教和用 药指导等，研究结果可能有助于发现 ATAAD 手术潜在的不良风险，以为您和和您 情况相似的其他受试者未来获得更高质量的医疗服务。

**11.您的权利**

是否参加研究完全取决于您的自愿。您可以在任何时候撤回知情同意而无需给出理 由。如果您决定退出研究（停止研究），必须通知您的主管医生；您的主管医生可能会询 问并记录下相关的健康状况及退出原因。

您可能不是完全明白这份知情同意书的内容，您可以向您的研究医生咨询，研究过

程中如有任何疑问，您也可以随时向您的研究医生咨询。

**12.参加本研究/试验的费用**

参加本试验无任何预期额外花费。

您不会因参加本项研究而获得任何报酬。

**13.隐私保密**

在试验过程中获得的关于您个人的任何信息和数据都将被严格保密。可以识别您身 份的信息将不会透露给研究小组以外的成员，除非获得您的许可。所有的研究成员和申 办方都被要求对您的身份保密。您的档案将保存在有锁的资料柜中，仅供研究人员查阅。 为确保研究按照规定进行，必要时，政府管理部门、申办方授权的监查员或伦理委员会 成员按规定可以在研究单位查阅您参加试验的相关信息，但他们将保证不向其他方泄露 您的信息。虽然研究结果可能会被发表，但不会在这些发表物中泄露您的身份。本研究 资料将保存在南京大学医学院附属鼓楼医院。

签署了这份书面知情同意书，即表明您已同意研究医生收集和处理您在本研究中的 个人信息（“研究数据”），包括：您的生日、性别、种族、生理和心理健康状况的个人 数据，除非撤回知情同意，否则意味着您的研究数据将一直可被采用。如果您撤回知情 同意，研究医生和申办方将不再利用您个人数据，但在撤回知情同意之前已经分享的个 人数据，仍可使用。

研究医生将使用研究数据进行临床研究。申办方可能将数据用于：进行临床研究， 支持研究药物上市许可的申请和研发新药产品、诊断或医疗辅助手段。

您拥有索取保存在研究医生和申办方的个人数据的权利，您同样拥有要求更正您个 人数据中不准确之处的权利；您有随时撤回知情同意的权利，如您有上述要求，请和研 究医生联系。

**14. 联系人信息**

◆如果您有进一步的问题，请联系：

医生姓名：	地址：	联系电话：

◆ 如果您有与受试者自身权益相关的问题，请联系南京大学医学院附属鼓楼医院医学 伦理委员会，电话：025-68182923

**知情同意书签字页**

受试者知情同意声明：

• 我已经阅读了这份知情同意书，已经获得关于此试验的背景、目的、试验步骤、风险 及获益情况，针对该临床试验的相关问题我有足够的时间和机会进行提问，并已得到满 意的解答。

• 我理解参加这项试验是自愿的。

• 我允许按知情同意书中所述使用和共享我的医疗信息。

• 我知道自己可以随时退出本试验而不会遭受利益损失或其他不利后果。

• 我愿意配合研究人员做相关的检查或者治疗。

• 我知道参加此项研究个人身份和隐私将被严格保密。

• 我也被告知，当我有问题或想进一步获得信息应当与谁联系。

• 我将获得一份已签字并注明日期的此知情同意书的副本。

受试者签字（印刷体）：	联系电话：

受试者签字（手写体）: 日	期:

监护人签字【 如适用 】( 印刷体 ，请注明和受试者直接 的关系 ) :

联系电话:

监护人签字(手写体 ) : 日	期:

执行知情同意的研究者声明：

我或我的研究团队已向该受试者充分解释和说明了本临床试验的背景、目的、试验 步骤、风险及获益情况，给予他/她足够的时间阅读知情同意书、与他人讨论， 并解答 了其有关研究的问题；我已告知该受试者当遇到问题时的联系方式；我已告知该受试 者（或监护人）他/她可以在研究期间的任何时候无需任何理由退出本研究。

研究者签字（印刷体）：	联系电话：

研究者签字（手写体）: 日	期:
